# Quality Control of Carbon Look Components via Surface Defect Classification with Deep Neural Networks

**DOI:** 10.3390/s23177607

**Published:** 2023-09-01

**Authors:** Andrea Silenzi, Vincenzo Castorani, Selene Tomassini, Nicola Falcionelli, Paolo Contardo, Andrea Bonci, Aldo Franco Dragoni, Paolo Sernani

**Affiliations:** 1 Dipartimento di Ingegneria dell’Informazione, Università Politecnica delle Marche, Via Brecce Bianche 12, 60131 Ancona, Italy; s1091549@studenti.univpm.it (A.S.); s.tomassini@pm.univpm.it (S.T.); n.falcionelli@pm.univpm.it (N.F.); p.contardo@pm.univpm.it (P.C.); a.bonci@univpm.it (A.B.); a.f.dragoni@univpm.it (A.F.D.); 2 HP Composites S.p.A., Via del Lampo S.N., Z.Ind.le Campolungo, 63100 Ascoli Piceno, Italy; v.castorani@hpcomposites.it; 3 Department of Law, University of Macerata, Piaggia dell’Università 2, 62100 Macerata, Italy

**Keywords:** quality control, carbon fiber reinforced polymers, CFRP, deep learning, transfer learning, Industry 4.0

## Abstract

Many “Industry 4.0” applications rely on data-driven methodologies such as Machine Learning and Deep Learning to enable automatic tasks and implement smart factories. Among these applications, the automatic quality control of manufacturing materials is of utmost importance to achieve precision and standardization in production. In this regard, most of the related literature focused on combining Deep Learning with Nondestructive Testing techniques, such as Infrared Thermography, requiring dedicated settings to detect and classify defects in composite materials. Instead, the research described in this paper aims at understanding whether deep neural networks and transfer learning can be applied to plain images to classify surface defects in carbon look components made with Carbon Fiber Reinforced Polymers used in the automotive sector. To this end, we collected a database of images from a real case study, with 400 images to test binary classification (defect vs. no defect) and 1500 for the multiclass classification (components with no defect vs. recoverable vs. non-recoverable). We developed and tested ten deep neural networks as classifiers, comparing ten different pre-trained CNNs as feature extractors. Specifically, we evaluated VGG16, VGG19, ResNet50 version 2, ResNet101 version 2, ResNet152 version 2, Inception version 3, MobileNet version 2, NASNetMobile, DenseNet121, and Xception, all pre-trainined with ImageNet, combined with fully connected layers to act as classifiers. The best classifier, i.e., the network based on DenseNet121, achieved a 97% accuracy in classifying components with no defects, recoverable components, and non-recoverable components, demonstrating the viability of the proposed methodology to classify surface defects from images taken with a smartphone in varying conditions, without the need for dedicated settings. The collected images and the source code of the experiments are available in two public, open-access repositories, making the presented research fully reproducible.

## 1. Introduction

In the fourth industrial revolution, commonly known as “Industry 4.0”, data-driven methodologies such as Machine Learning (ML) and Deep Learning (DL) are recognized essential for the implementation of production systems capable of self-organizing, predicting (and correcting) their own faults, and adapting to variable human needs [1,2]. ML and DL are enabling several Industry 4.0 applications, such as predictive maintenance [3], anomaly detection [4], aided design [5], and many others [6].

A prominent example of an Industry 4.0 application is the automatic control of the quality of products and their components [7]. Indeed, defect detection technology is capable of working in the long run with high precision, with obvious advantages over manual quality control [8]. In addition to the quality control of the final product, as in plastic injection molding [9,10], the detection of defects in the materials used in manufacturing is considered pivotal in many application domains, from the reliability of aircrafts to the efficacy of sanitary systems [11]. In this regard, the available literature focuses on integrating traditional Nondestructive Testing (NDT) techniques, such as Infrared Thermography [12], with ML and DL methodologies, in order to detect structural and inner defects that might compromise the integrity of the composite materials.

Instead, this paper studies the use of deep Convolutional Neural Networks (CNNs) to classify surface defects in composite materials and specifically Carbon Fiber Reinforced Polymers (CFRP) for the automotive sector, using plain images of real defective parts, without integrating any NDT techniques. Given the difficulty of obtaining analytic models to automatically detect defects of CFRP-covered components, due to their complex structure, and the challenge of standardizing characterization and measuring of such defects [13], neural networks can help in addressing the challenge by being used in the quality control of such components [14]. The images used in the experiments presented in this paper come from the production of CFRP of “HP Composites Spa”, a company located in Ascoli Piceno, Italy, and specialized in providing carbon fiber reinforced components. Specifically, in “HP Composites Spa”, the quality control of carbon look components covered with CFRP is performed with the naked eye by specialized staff to check for surface defects such as weft discontinuities and porosities that might endanger the aesthetics of the components. Hence, we want to understand if deep CNNs are suitable to automate or integrate the quality control by automatically processing plain images of carbon fiber reinforced components. As we are interested in the appearance of carbon look components rather than the structural performance (which has been extensively covered in the literature, such as in pultrusion [15]), in this study, we aim at understanding whether a transfer learning approach is adequate to classify the surface defects from smartphone pictures of the components. Therefore, we do not apply any NDT techniques requiring dedicated settings, usually implemented to detect structural or inner defects of components made of composite materials [16,17,18,19]. In this regard, transfer learning is suitable as it implies the use of models already trained on a large dataset to execute a different classification task, i.e., in a different feature space [20].

The research work performed on the “HP Composites Spa” case study and described in this paper adds the following contributions to the state of the art of surface defect detection in composite materials:

- A new database composed of plain images of CFRP-covered components for the automotive sector is introduced. The database is intended to train and benchmark techniques to classify defective and non-defective parts, as well as for multi-class classification into non-defective, recoverable, and non-recoverable components, using the pictures only, without thermography or any other NDT techniques. The database includes 400 images (200 per class) intended for the binary classification task and 1500 images (500 per class) for the multi-class classification. All the images are 224 × 224 pixels (96 ppi) in a JPEG format. The database is publicly released in an open-access GitHub repository (the image database is publicly available at: https://github.com/airtlab/surface-defect-classification-in-carbon-look-components-dataset, accessed on 10 July 2023).
- A systematic comparison of ten models for the classification of surface defects is provided. The models are deep neural networks based on ten pre-trained Convolutional Neural Networks (CNNs), implemented to process the samples end to end, testing the effectiveness of transfer learning and fine tuning in the classification of the surface defects of carbon look components. Specifically, the tested pre-trained CNNs are VGG16 and VGG19 [21], ResNet50 version 2, ResNet101 version 2, and ResNet152 version 2 [22], Inception version 3 [23], MobileNet version 2 [24], NASNetMobile [25], DenseNet121 [26], and Xception [27]. The CNNs are combined with fully connected layers, trained from scratch on the proposed dataset. The source code of the comparison is publicly available in a GitHub repository (the source code of the experiments is publicly available at: https://github.com/airtlab/surface-defect-classification-in-carbon-look-components-using-deep-neural-networks, accessed on 10 July 2023).
- A real application of Industry 4.0 is demonstrated, proposing the use of DL to automate the control of surface defects of carbon look components.

The main innovation introduced by the proposed approach is the possibility of avoiding the dedicated settings to detect and classify surface defects as those required by the ML and DL techniques available in the related literature. In this regard, the significance of the proposed image database with respect to the literature relies in its use to train and test techniques to detect surface defects on plain images, without the use of NDT techniques. In addition, as it is based on CNNs that exploit transfer learning, the proposed approach has the potential to be integrated in handheld devices to run the classification with the trained models. In this way, the assessment of the aesthetics of the carbon look components would be automated and standardized, without requiring complex skills. Furthermore, to the best of our knowledge, this is the first work in surface defect classification for composite materials to publicly release the data and the source code of the tests, providing reproducible results. To this end, it is worth noting that while the weights of the pre-trained networks are available and linked in the source code repository, we do not provide the weights of the fine-tuned models. In fact, our experiments are based on a stratified shuffle-split cross-validation strategy, where the networks are fine-tuned ten different times on diverse data, averaging the results got on the test sets. However, we do provide the code to run the cross-validation, making our experiments fully reproducible.

The rest of the paper is organized as follows. Section 2 presents a literature review about the use of deep learning for the detection of defects in composite materials, comparing our methodology to some related research works. Section 3 describes the database built for the experiments and the methodology followed to detect surface defects in the CFRP for the automotive sector. Section 4 presents the experimental evaluation of the surface defect detection applied to the CFRP used in HP Composites Spa, discussing the results. Finally, Section 5 draws the conclusions of this research.

## 2. Related Works

Nondestructive Testing (NDT) includes a multitude of techniques for the evaluation of materials and components without causing damage [28]. Therefore, even the properties of composite materials are evaluated with different NDT techniques, such as Infrared Thermography, Ultrasonic Testing, Radiographic Testing, and many others [29].

Following the effectiveness demonstrated in many application fields, such as image processing, object detection, speech recognition, and pattern recognition in general [30], DL is being integrated with NDT techniques, especially for the detection of defects that might jeopardize the reliability of composite materials. To this end, therefore, we present the works which are the state of the art of the integration of DL and NDT techniques, and, as such, achieved the best performance. For example, Liu et al. [17,31] proposed to use DL to improve the existing Infrared Thermography techniques. Specifically, they use Generative Adversarial Networks (GAN) as an image augmentation approach to enhance thermographic images in order to detect defects via Principal Component Analysis (PCA). They tested their methodology on specimens fabricated with intentionally formed defects. Differently, Bang et al. [16] suggested to use DL to directly detect defects by using Faster Region-based Convolutional Neural Network (Faster RCNN) on thermographic images. Specifically, they trained and validated an Inception V2 CNN [32] on 2802 thermographic images, obtained via data augmentation from 467 original images downloaded from the Internet. Then, they obtained a 75% average precision on 320 images of two composite specimens (carbon fiber fabric and randomly oriented glass fiber) with artificial defects. A similar study was conducted by Fang et al. [18] who used Mask-RCNN, an extension of Faster CNN, on a dataset composed of 500 images of two different materials, plexiglass and CFRP. They achieved an 86.2% average accuracy in detecting defects. Wei et al. [19] developed a U-Net [33] variant to segment damages on around 8000 thermographic images, achieving the best F1 score of 92.74% in detecting the simulated defects on Carbon Fiber Reinforced Polymers (CFRP). Marani et al. [34] applied a CNN on seven videos (with various lengths) generated by Step Heating/Long Pulse Thermography on Glass Fiber Reinforced Polymers (GFRP). They achieved an average accuracy of 84.6% in classifying four defect classes. Of course, Infrared Thermography is not the only NDT technique combined with DL. For example, Meng et al. [35] applied DL on ultrasonic signals, developing a CNN to classify defects on CFRP laminates. Their CNN-based classifier performed ultrasonic pattern recognition on 1000 samples produced on purpose (5000 were used for training) and was able to classify 10 defect classes with a 98% recognition rate. Gong et al. [36] applied a CNN to X-ray images to detect slag inclusions in aeronautics composite materials. However, due to the lack of defective components to include in the dataset, they trained their CNN on different data (using X-ray images of welded components) and then applied the trained model to the detection of slag inclusion defects in aeronautics composite materials. They achieved a 96.8% accuracy on 260 testing samples.

Despite the use of different techniques to detect defects in diverse materials, the described related works share some common traits:

- They apply DL on top of NDT techniques, such as Infrared Thermography, to detect structural and inner defects inside the composite materials.
- Most of the described works [16,17,31,35] record accuracy metrics in the detection of defect on specimens with artificial damages, produced on purpose.
- They do not publicly release the data and the source code of the experiments performed to collect the accuracy metrics.

The methodology proposed in this paper differs from the described works on these points. Specifically, we identify components that might compromise the aesthetics of the carbon fiber reinforced fabric by directly applying deep neural networks on images, without using any specific NDT technique. In fact, the suggested methodology circumvents the necessity for specific configurations needed by the NDT techniques. This characteristic distinguishes it from described related works. Combining DL with NDT techniques requires tailored settings to identify and categorize defects in CFRP components. These specialized configurations can be resource-intensive, requiring precise tuning of parameters and advanced expertise to set up properly. Our proposed approach simplifies this process, enhancing the accessibility and applicability of automated quality assessment in CFRP manufacturing. Therefore, in our work, we focus on surface defects in composite materials, following the results achieved, in general, in surface defect detection in other materials. In fact, DL achieved outstanding results in defect recognition [37] and has been successfully applied to the detection of surface defects in different materials, for example, on metal [38], steel [39,40], fabric [41,42], and wood [43,44]. Moreover, we evaluated our models on images of real defective parts instead of introducing artificial defects. With respect to the works that use artificial defects to test their methodologies, in our work, we use images of real defects, trying to cope with the level of complexity and randomness that artificial defects might fail to replicate. They can vary greatly in shape, size, location, and context, unlike synthetic defects, which are usually standardized and less representative of the true range of possible flaws. By training and evaluating our models on real defective parts, we address the capacity to accurately recognize and classify an array of real-world defects, enhancing the robustness and practical utility of the classification models.

The proposed image database also reflects the listed differences with the related works. It contains real-world images of defective and non-defective components, with no synthetic data; in addition, it is composed of plain images only, i.e., it does not include images collected with thermal cameras or in other dedicated settings typical of NDT techniques. Moreover, we released the images included in the proposed database and the source code of experiments in two public open-access GitHub repositories, making our experiments fully reproducible.

## 3. Materials and Methods

The goal of this research is to understand whether we can classify surface defects in components made of composite materials (specifically those covered with CFRP) using deep neural networks on plain images without applying any NDT techniques with dedicated settings. In fact, the aesthetics of carbon look components are essential in the automotive sector, to the point that, in “HP Composites Spa”, the quality of such components is manually checked with the naked eye by specialized operators.

With the aim of investigating whether deep neural networks can be successfully applied to images of surface defects taken with a smartphone, we built a database of plain images of carbon look components intended to benchmark classifiers of such surface defects. The database was split into two datasets to test both binary classification (“defect” vs. “no defect”) and multi-class classification, distinguishing between defect types. To perform such classifications, we proposed an end-to-end classifier architecture using deep neural networks and transfer learning, comparing ten different pre-trained CNNs as feature extractors. To this end, Section 3.1 and Section 3.2 describe the proposed database of images and deep neural network architecture.

### 3.1. Proposed Image Database

**Figure 1:**
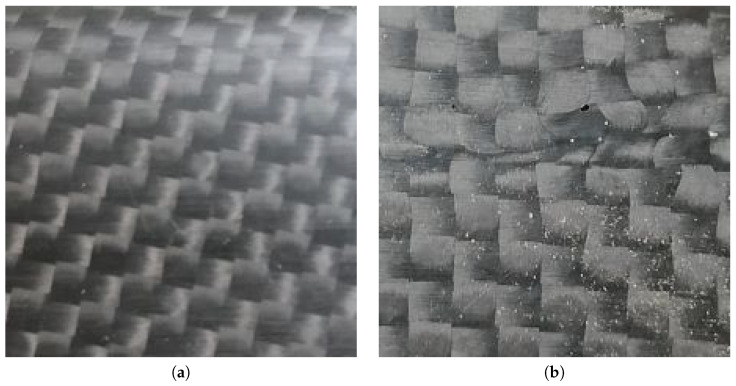
Negative (**a**) and positive (**b**) samples from the binary dataset. The negative sample includes no defect. Instead, the positive sample has a weft discontinuity at the center and porosities in the carbon fiber reinforced fabric covering the component.

**Figure 2:**
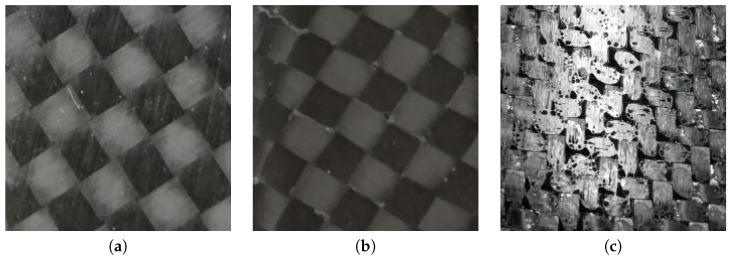
Negative (**a**), recoverable defect (**b**), and non-recoverable defect (**c**) class samples from the multi-class dataset. The negative samples has no defects. The recoverable defect sample includes spare porosities. The non-recoverable defect sample includes severe porosities and weft discontinuities.

To evaluate the proposed end-to-end classifiers of surface defects, we built a database of plain images of the carbon look components produced in “HP Composite Spa”. The images are 224 × 224 pixels (96 ppi) in the JPEG format. The images were originally taken at 3968 × 2976 pixels, then cropped at the center and resized at 224 × 224 to cope with the input size of the pre-trained CNNs used in this study. Moreover, the images depict components with carbon covered flat surfaces or with a slight curvature. The database is split into two datasets:

- One dataset is binary, including two classes. A total of 200 images are labeled as “no defect” (Figure 1a), as they are with no defects or present limited recoverable porosities; 200 images are labeled as “defect” (Figure 1b), as they include weft discontinuities. This set of images is intended for binary classification, to test the performance of models that sort the components into defective or non-defective.
- The second dataset is multi-class, including three classes, i.e., “no defect”, with recoverable defects, and with non-recoverable defects. The dataset contains 1500 images, 500 per class. The “no defect” class (Figure 2a) includes images of components without any surface defect. The recoverable defect class (Figure 2b) includes images of components with limited porosities and the infiltration of external materials (such as aluminum). Such defects can be treated and corrected. Finally, the non-recoverable defect class (Figure 2c) includes images of components with weft discontinuites, severe porosities, and resin accumulations. In “HP Composite Spa”, these components are discarded, as their appearance cannot be recovered. With such dataset, we test the capability of the proposed models to classify multiple defect classes.

Specifically, the recoverable defects are:

- Isolated porosities, i.e., isolated holes in the surface of the material that only damage the aesthetic performance, but not the structural tightness (an example is provided in Figure 2b).
- Infiltration of foreign objects (aluminum or polyethylene) on the material surface that can be removed.

Instead, the non-recoverable defects are:

- Severe porosities, i.e., where the holes in the surface of the material are not isolated and cover most of the surface (an example is provided in Figure 2c).
- Weft discontinuities, i.e., all the cases in which the characteristic texture of the interwoven carbon fiber bundles is altered, generally caused by a wrong overlapping of the materials or poor fiber adhesion to the mold (an example is provided in Figure 1b).
- Accumulations of resin caused by the imperfect calibration of the spaces between the two fiber molds and the silicone mandrel interposed between them.

The images are real-world examples of all the surface defects that “HP Composites Spa”, the company that provided the images, need to classify.

The images were manually labeled according to the recommendations of the domain experts, i.e., the HP Composite staff, in particular to distinguish between recoverable and non-recoverable defects. The images were taken from different angles and distances, without standard lighting. In this way, we emulated the conditions that an operator, in charge of controlling the appearance of the components, would face using a handheld device for the quality control, without dedicated settings as in NDT. Thus, we investigated the capability of the proposed models of working under varying conditions in order to assess the viability of our approach based on plain images taken in a non-standard, non-fixed environment.

We trained and tested the models proposed in this paper on both datasets, in their original form. Moreover, we ran the classification tests on an augmented version of the datasets. In fact, despite the fact that 400 images and 1500 images are available to test the classification with two classes and three classes, respectively, deep neural networks usually require a higher number of training images to reduce the classification error. To face such issue, data augmentation has been proven useful to cope with the scarcity of image data and overfitting problems [45]. To this end, in our experiments, we augmented the datasets by flipping the original images on both axes. Thus, we built an augmented binary dataset composed of 1200 images (600 per class): 400 are the original images, 400 are the same images, but flipped on the horizontal axis, and 400 are flipped on the vertical axis. Similarly, we built an augmented multi-class dataset composed of 4500 images (1500 per class). We applied flipping (i.e., a 180${}^{\circ }$ rotation on both axis) without using other augmentation techniques (such as rotation and scaling) to check the capability of the proposed model to converge to a proper training with a relatively small set of data for fine-tuning. In addition, adding other augmentation techniques to the collected dataset could have caused overfitting in the models, as it would have increased the replication of the same data.

### 3.2. Proposed Classification Model

**Figure 3:**
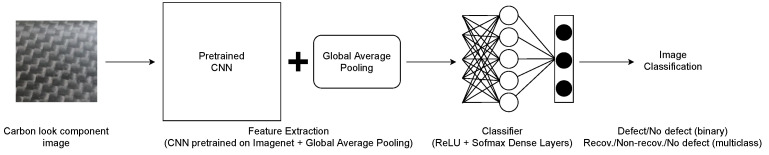
The architecture proposed to classify the plain images of carbon look components. The input is processed by a CNN pre-trained on the Imagenet database. The CNN, without its final fully connected layers, is combined with a Global Average Pooling layer and used to extract features. Then, fully connected layers are added and trained from scratch on the dataset. The final classification is performed by the last dense layer, with the Softmax activation function. Different combinations of fully connected layers (and hyperparameters) have been tested to improve the final classification (see Table 1 and Table 2).

Figure 3 shows the architecture of the end-to-end models proposed in this paper. To classify the plain images of carbon look components, we compared ten models based on ten deep CNNs pre-trained on the Imagenet database [46] used as a feature extractor, combined with a classifier based on fully connected dense layers. In fact, we applied a transfer learning methodology, i.e., the use of models already trained on a large dataset to execute a different classification task. In addition to dealing with the scarcity of training data, transfer learning can achieve better generalization than a dedicated training from scratch and prevent overfitting [47,48]. In this regard, transfer learning based on the pre-training on ImageNet has been recognized as particularly effective for general-purpose classifiers [49]. While the database proposed in this paper is too small to train the CNNs from scratch, training from scratch on images of surface defects of carbon look components would be feasible, at the risk of introducing overfitting. Therefore, we compared ten different pre-trained CNNs, namely VGG16, VGG19, ResNet50V2, ResNet101V2, ResNet152V2, InceptionV3, MobileNetV2, NASNetMobile, DenseNet121, and Xception. We removed from the pre-trained CNNs the fully connected layers, averaging the output of the last convolutional layers with Global Averaging Pooling. The obtained feature vector was used as the input for fully connected dense layers composed of neurons with the Rectified Linear Unit (ReLU) activation function. The final classification was performed by a dense layer using the Softmax activation function, composed of two neurons for the classification on the binary dataset, and three neurons for the multi-class dataset.

We trained each model in an end-to-end fashion on the proposed datasets, fine-tuning the pre-trained CNNs and training the fully connected layers from scratch. Specifically, we looked for the best hyperparameters in terms of classification accuracy on the binary dataset and the multi-class dataset. We tuned the following parameters:

- The number of the CNN final layers to be fine-tuned on the proposed dataset, testing 8, 4, and 0. This means that, during the end-to-end training of the model, the weights were freezed for the starting layers of the pre-trained CNNs, while for the last 8 (or 4 or none) layers, the weights were modified with the backpropagation.
- The optimizer to perform the error backpropagation, testing the Stochastic Gradient Descent (SGD) with a 0.9 momentum and Adam. For both, we compared different learning rates, i.e., 0.001 and 0.0001.
- The use of Batch Normalization for regularization between the Global Average Pooling and the first dense layer.
- The number of fully connected layers to be added to the pre-trained CNNs to perform the final classification. Specifically, we tested a single dense layer composed of 512 ReLU neurons followed by a final layer with Softmax activation, and two dense layers composed of 256 and 128 ReLU layers, followed by the Softmax layer.

To test the impact of the hyperparameters on the classification performance, we used the same evaluation protocol described in Section 4.1, based on a stratified shuffle split cross-validation repeated ten times. For all the models, we used the same batch size, i.e., 32 images, and the same number of training epochs, i.e., 100, applying an early stopping strategy after 5 epochs without lowering the validation loss to avoid the overfitting.

To this end, Table 1 lists the configurations of hyperparameters which offered the best results in terms of classification accuracy among those tested on the binary dataset, including the fully connected layers used for classification and trained from scratch on the proposed dataset. In most of the models, the last 8 layers of the pre-trained CNNs were fine-tuned. Instead, the model based on MobileNetV2 offered its best results by freezing the weights of all the layers in the pre-trained CNN, training only the dense layers added for the final classification. With NASNetMobile and Xception, only the last 4 layers were fine-tuned. The models based on VGG16, VGG19, and InceptionV3 offered the best classification accuracy using the SGD optimizer for training. Instead, the other seven models obtained the best performance with Adam. The Batch Normalization did not add any improvement on the classification accuracy. Finally, the models based on MobileNetV2, NASNetMobile, and Xception offered the best results using two consecutive dense layers with 256 and 128 ReLU neurons. The other seven models obtained the best metrics with the dense layer composed of 512 ReLU neurons.

Table 2 includes the combinations of hyperparameters with the best results in terms of classification accuracy on the multi-class dataset. All the models achieved the top accuracy by fine-tuning the last 8 layers of the pre-trained CNNs and using a single dense layer with 512 ReLu neurons. The Batch Normalization improved the results for the models based on VGG16 and VGG19. These two models and the one based on MobileNetV2 used the SGD optmizer for the training, whereas the other seven models obtained the best metrics with Adam.

## 4. Experimental Evaluation

We compared the ten proposed end-to-end deep neural networks on the collected image database, evaluating accuracy metrics for the binary and the multi-class classification. In this section, we report the details about the results achieved by the best configuration (i.e., the set of hyperpameters, among those tested, which achieved the best accuracy) of each classifier, as explained in Section 3.2. We aim at understanding whether the pre-trained CNN-based networks can be used to detect and classify the surface defects of carbon look components, in the HP Composite case study and in general. Indeed, in addition to the creation of a baseline of metrics on the collected images, a comparison of classifiers might be relevant to other case studies about surface defect detection and classification in composite materials.

Therefore, in the following subsections, we describe the experimental setup as well as the metrics (Section 4.1) evaluated to compare the classifiers. Moreover, we present and discuss the results of the evaluation (Section 4.2). Finally, we analyze the limitations of our experimental evaluation (Section 4.3).

### 4.1. Evaluation Protocol and Metrics

We tested the proposed end-to-end deep neural networks on the binary and multi-class datasets, using both the original version and the augmented one, by applying a stratified shuffle split cross-validation scheme. In this regard, we repeated a randomized 80-20 split ten times, using the 80% of the data as the training set, and the 20% as the test set, preserving the percentage of samples from each class, in each split. A total of 12.5% of the training images, i.e., 10% of the whole dataset, was used as validation data in each split. To implement a fair comparison, the splits were the same for all the tested models. In this way, we tested the generalization capability of our models, making sure that they did not overfit on a specific data split. Table 3 includes the number of training, validation, and test images for each split of the described cross-validation scheme.

As highlighted in the Section 1, a Jupyter notebook with the described experiments is available in a public GitHub repository in order to guarantee the reproducibility of the tests. The experiments ran on a virtual machine in a cloud environment, equipped with an 8-core Intel Xeon CPU E5-2623 v4 (2.60 GHz) (Intel Corporation, Santa Clara, CA, USA), 30 GB of RAM, and a Nvidia Quadro M4000 GPU (Nvidia Corporation, Santa Clara, CA, USA), using Keras 2.6.0, TensorFlow 2.6.0, and scikit-learn 1.0.2.

We compared the proposed end-to-end networks by measuring the average testing accuracy over the ten splits of the cross-validation scheme. Therefore, in each iteration, we computed the ratio between the samples which are correctly classified and the total number of samples in the test set. Moreover, we aggregated the results in each split to compute:

- The precision for each class, i.e., the ratio between the number of samples correctly classified as belonging to a class and the total number of samples labeled as that class in the test set.
- The recall for each class, i.e., the ratio between the number of samples correctly classified as belonging to a class and the total number of samples available for that class in the test set.

Precision and recall can be formulated in terms of true positives (TP), true negatives (TN), false positives (FP), and false negatives (FN) according to the following equations:

(1) $$\begin{array}{cc} precision & =\frac{TP}{TP+FP}, \end{array}$$

(2) $$\begin{array}{cc} recall & =\frac{TP}{TP+FN}. \end{array}$$

In the binary classification, to compute the precision and recall of a class, the samples of that class are considered positive, whereas the samples of the other class are considered negative. In the multi-class classification, precision and recall for a *C* class generalize to the following equations:

(3) $$\begin{array}{cc} precision_{C} & =\frac{T_{C}}{T_{C}+F_{C}}, \end{array}$$

(4) $$\begin{array}{cc} recall_{C} & =\frac{T_{C}}{T_{C}+F_{¢}}, \end{array}$$

where $T_{C}$ is the number of samples correctly classified as *C*, $F_{C}$ represents the number of samples labeled as *C* but actually belonging to a different class, and $F_{¢}$ is the number of samples actually belonging to *C* but labeled with a different class.

Finally, we report the Receiver Operating Characteristic (ROC) curve and the Area Under the Curve (AUC) for the best model on both dataset to provide a complete picture of the diagnostic capabilities of the end-to-end networks. Considering the “no defect” as the negative class and the “defect” as the positive class, the ROC curve shows the true positive rate (TPR, i.e., the recall of the “defect” class) against the false positive rate (FPR, i.e., 1 minus the recall of the “no defect” class) when the classification threshold varies, for all the splits of the cross-validation. In the multi-class classification, the ROC curve is computed micro-averaging the ROC curves obtained for each label by binarizing the output (i.e., by considering one label as positive and all the other as negative).

### 4.2. Results and Discussion

In this subsection, we analyze the metrics obtained by the proposed neural networks on the collected image database, discussing the results on the binary classification task (“no defect” vs. “defect”) and the multi-class classification task (“no defect” vs. “recoverable defect” vs. “non-recoverable defect”). We also report on the impact of the data augmentation on the accuracy of the proposed neural networks.

#### 4.2.1. Results on the Binary Classification Task

Table 4 includes the class precision and recall obtained by the end-to-end networks based on different pre-trained CNNs on the original binary dataset. The metrics are averaged across the ten splits of the stratified shuffle split cross-validation scheme. With 140 training images per class, the end-to-end networks struggle to converge to a good classification result for both classes, given that precision and recall are below 90% for all the models. In fact, the results vary across different data splits, with ResNet101V2 obtaining the highest standard deviation in the recall of the “no defect” class (17.64%). For example, in one of the splits, the network based on ResNet101V2 wrongly classifies 24 “no defect” samples out of 40 as containing defects, achieving a recall of 35%, whereas all the 40 “defect” samples are correctly classified, with a 100% recall. This means that, on such data split, the network tends to label most of the samples as defective. On the contrary, in a different data split, the ResNet101V2-based model correctly classifies 39 out of 40 “no defect” samples (recall 97.5%), but wrongly labels 30 out of 40 “defect” samples (recall 25%), tending to label the samples as “no defect”. The best classifier is the network based on Xception, achieving the highest recall (87.64%) for the “no defect” class, and the highest precision (87%) and recall (89.75%) for the “defect” class. Xception also obtains the second-best precision (89.57%) for the “no defect” class, with the lowest standard deviation (2.69%). This means that the Xception-based model is capable of correctly classifying around 90% of the testing samples, independently from the label. The second-best model is the one based on DenseNet121, obtaining scores which are two percentage points below those of Xception. The other models achieve lower precision and recall, with higher standard deviation.

Therefore, the results on the original binary dataset do not support the idea of classifying plain images of carbon look components into two classes, i.e., with defect and with no defect, at least when few training samples are available. With 140 training images per class, transfer learning is not effective for such binary classification task.

However, a significant improvement in the collected metrics occurs with data augmentation. To this end, Table 5 lists the average values of class precision and recall computed on the augmented binary dataset. For example, the recall of the “no defect” class increases from 60% ± 17.6% to 94 ± 3.1% for the model based on ResNet101V2. In general, all the networks are much more stable, with standard deviations significantly lower than those obtained on the original dataset.

**Figure 4:**
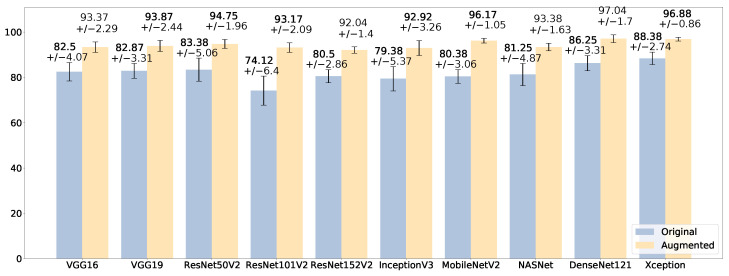
Comparison of the average accuracy (%), with standard deviation, on the original binary dataset (blue) and the augmented one (yellow).

**Figure 5:**
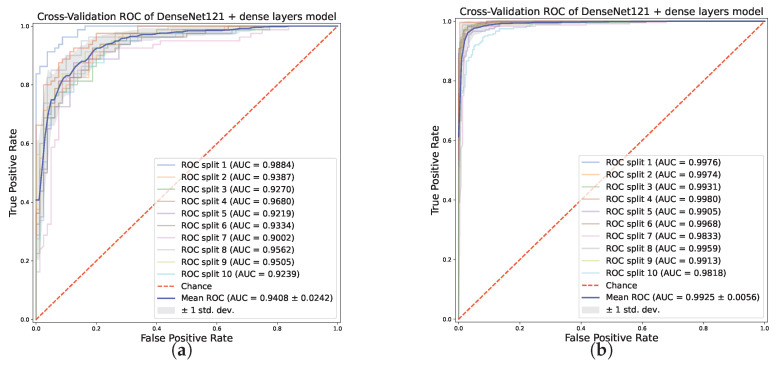
ROC curve for each split of the stratified shuffle split cross-validation scheme for the DenseNet121-based model, on the original binary dataset (**a**) and the augmented one (**b**).

The impact of data augmentation is evident in Figure 4, which reports the average accuracy (and its standard deviation) of each model across the ten splits of the cross-validation, in the original and in the augmented binary datasets. The best model for the binary classification task on the augmented dataset is the one based on DenseNet121, achieving a 97.04% (±1.7) average accuracy, increasing by 11% its performance obtained on the original dataset. Xception scores a similar average accuracy (96.88%) but with a lower standard deviation (0.86%). MobileNetV2 also obtains a 96.17% accuracy (±1.05%). The model based on ResNet101V2 is the one with the biggest improvement from the original dataset to the augmented one, improving from a 74.12% (±6.4%) average accuracy to 93.17% (±2.09%). In addition, the impact of data augmentation is highlighted by the ROC curve reported in Figure 5a for the original dataset and in Figure 5b for the augmented dataset, for the DenseNet121-based model. In the augmented dataset, the curve shows a lower variance, i.e., a greater stability of the classifier across the ten splits of the stratified shuffle split cross-validation. The average AUC is also greater (99.25 ± 0.56%) than that of the original dataset (94.08 ± 2.42%).

Therefore, the results achieved on the augmented dataset allow concluding that the classification of plain images into containing defects or not is feasible from smartphone images. The results are promising, as the best models (DenseNet121, Xception) achieve around 97% accuracy across all the splits of the cross-validation scheme.

#### 4.2.2. Results on the Multi-Class Classification

The results on the multi-class classification exhibit a trend similar to those of the binary classification: the classification accuracy improves when using the augmented dataset instead of the original one. To this end, Table 6 shows the average class precision and recall on the original multi-class dataset, computed across the ten splits of the stratified shuffle split cross-validation scheme. All the ResNet-based models cannot converge to a proper classification on all the classes, interpreting as “recoverable” or “non-recoverable” images with no defect. Their classification capability strongly depends on the way data are randomly split. For example, ResNet50V2 exhibits a 21.20% standard deviation on the 61.60% recall for the “no defect” class. On the original dataset, the models which seem to converge to the classification of samples are those based on VGG16, VGG19, and DenseNet121 with the precision and recall between 86% and 93% for each class. Instead, all the other models obtain lower metrics.

The classification capability of the proposed models significantly improves when using the augmented dataset, as shown in Table 7. The models based on DenseNet121, VGG16, and VGG19 obtain more than 95% of precision and recall for all the classes. In general, all the models display better performance than the original dataset.

**Figure 6:**
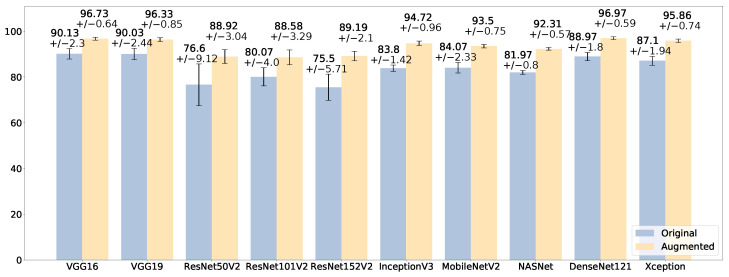
Comparison of the average accuracy (%), with standard deviation, on the original multi-class dataset (blue) and the augmented one (yellow).

**Figure 7:**
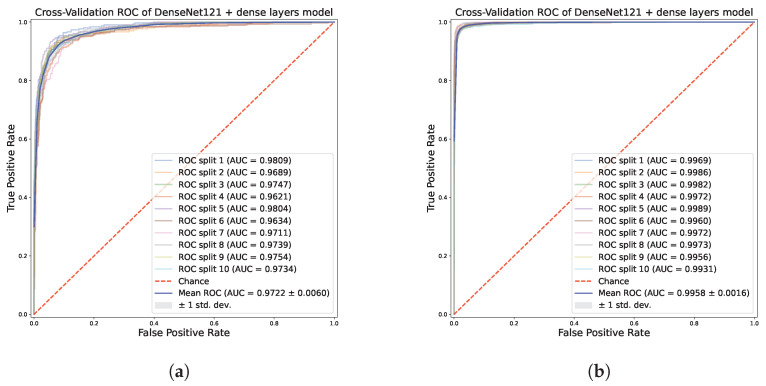
ROC curve for each split of the stratified shuffle split cross-validation scheme for the DenseNet121-based model, on the multi-class dataset (**a**) and the augmented one (**b**).

The good performance with the augmented dataset, in comparison with the original one, is evident in Figure 6, which reports the average accuracy across the ten splits of the cross-validation scheme. The best model in the multi-class classification is the one based on DenseNet121, which has the best average accuracy, 96.97%, and the lowest standard deviation, 0.59%. The models based on VGG16 and VGG19 are very close, with 96.73 (±0.64) and 96.33% (±0.85) respectively. The model based on Xception, which was the second best in the binary classification, also exhibits a promising accuracy, scoring 95.86% (±0.74). Hence, in addition to very good accuracy, the low standard deviation highlights that these models are independent from the data splits of the cross-validation, demonstrating their generalization capability. The good impact of data augmentation on the classifiers is depicted in Figure 7, which includes the ROC curves across the cross-validation splits of the model based on DenseNet121 on the multi-class dataset. The diagnostic capability of the classifier clearly increases in the augmented dataset, with the curves tending to the upper left corner of the diagram independently from a specific data split.

These results allow concluding that classifying surface defects of carbon look components from plain images is definitely possible and transfer learning can be applied; different deep neural networks based on pre-trained CNNs are able to classify 900 images into recoverable, non-recoverable or those with no surface defects. The simplicity of the proposed models and the good accuracy achieved on images taken from different points of view, with varying lighting, suggest that handheld devices could be used in practice, in addition to the naked-eye quality control of carbon look components currently performed in the company that provided the data for our experiments. In this way, a member of staff could spot the defect and use the proposed methodology to classify it in a standardized fashion. In fact, such architectures can be deployed in mobile devices or in the cloud and used to process smartphone pictures during quality control.

### 4.3. Limitations

The results are promising both in terms of binary classification (“defect” vs. “no defect”) and multi-class classification (“recoverable” vs. “non-recoverable” vs. “no defect”), but present some limitations. The proposed datasets include 400 images for the binary classification task and 1500 images for the multi-class classification task. Data augmentation solves the scarcity of training images and is, in general, recognized useful to limit overfitting. However, testing with more original images of diverse components would allow drawing more general conclusions. This limitation can be addressed by collecting even more real-world images to expand the dataset and furtherly limit overfitting. Nevertheless, to the best of our knowledge, the database released with this paper is the first publicly available set of plain images of surface defects in carbon look components.

Furthermore, the collected dataset contains real-world images, with realistic noise (all the images were acquired with a smartphone) and include all the defects relevant to the company that has been our case study. In this regard, our study focused on surface defects. Indeed, other applications might include different type of defects (including structural ones) and more noise. Therefore, to obtain more general results and detect different type of defects, the dataset should be expanded and a different training run. Moreover, the image resolution (224 × 224 pixels) and the collection of images in a real-world scenario, with a smartphone without standardizing the point of view, show the capability of our classifiers in low-quality conditions. However, to obtaion more general results about the robustness of the proposed models with even lower quality images, the dataset can be further expanded by adding artificial blur and noise to the images.

Finally, the results suggest that the proposed classifiers could be added to the naked-eye quality control of carbon look components currently performed in the company that was our case study. However, before proceeding to production, a study dedicated to verifying computation times and the image processing on handheld devices might be necessary in order to draw definitive conclusions in this regard. Furthermore, we obtained the presented results by testing different combinations of hyperparameters of the proposed models. Those based on DenseNet121 and Xception for the binary dataset, and DenseNet121 and VGG16 for the multi-class dataset, showed the best generalization capability. A more detailed investigation about the best hyperparameters specifically dedicated to such models might be worth conducting to find the optimal combination.

## 5. Conclusions

In this paper, we presented a comparison of ten end-to-end neural networks based on pre-trained CNNs, developed to classify surface defects in carbon look components (covered with CFRP) in a transfer learning fashion. We compared the proposed networks on a database of images from a real case study, with pictures of carbon look components for the automotive sector taken with a smartphone, under varying lighting conditions and from different points of view. In this way, we studied the use of the classification of plain images as a tool to automate or integrate a task currently performed by specialized operators with the naked eye. Our approach differs from other methodologies available in the literature as it does not use NDT techniques requiring dedicated settings, such as Infrared thermography.

The results are promising: the best model, the one based on DenseNet121, is capable of distinguishing between 900 images of components with recoverable defects, non-recoverable defects and those without any defect with a 97% accuracy. The other three models, those based on VGG16, VGG19, and Xception, exhibit a similar accuracy. Although the generality of such accuracy should be interpreted with caution due to the reasons explained in Section 4.3, the proposed end-to-end deep neural networks are adequate to classify surface defects in the carbon look components produced in “HP Composite Spa”. The simplicity of the proposed methodology, based on end-to-end deep neural networks, and the classification accuracy with images taken from different points of view, with varying lighting conditions, make the quality control of carbon look components feasible. Given the possibility of porting CNNs into mobile devices [50,51], our approach can be integrated into handheld devices by deploying the classifier locally or in the cloud. Nevertheless, as highlighted in Section 4.3, a study dedicated to verifying computation times and the image processing on handheld devices might be necessary in order to draw definitive conclusions in this regard.

The source code of the experiments, including the ten end-to-end networks, and the database of images collected for the tests are publicly available in dedicated open-access repositories. The database of images can be useful to benchmark other classification techniques. The source code of our experiments makes the research presented in this paper fully reproducible.

## Figures and Tables

**Table 1:** The hyperparameters and the final layers of each model which obtained the best results on the binary augmented dataset.

	Fine-Tuning	Optimizer	Learning Rate	Batch Normalization	Dense 512 Units	Dense 256 Units	Dense 128 Units
**VGG16**	Last 8	SGD	0.0001	-	✓	-	-
**VGG19**	Last 8	SGD	0.001	-	✓	-	-
**ResNet50V2**	Last 8	Adam	0.0001	-	✓	-	-
**ResNet101V2**	Last 8	Adam	0.001	-	✓	-	-
**ResNet152V2**	Last 8	Adam	0.0001	-	✓	-	-
**InceptionV3**	Last 8	SGD	0.001	-	✓	-	-
**MobileNetV2**	0	Adam	0.001	-	-	✓	✓
**NASNet**	Last 4	Adam	0.0001	-	-	✓	✓
**DenseNet121**	Last 8	Adam	0.0001	-	✓	-	-
**Xception**	Last 4	Adam	0.0001	-	-	✓	✓

**Table 2:** The hyperparameters and the final layers of each model which obtained the best results on multi-class augmented dataset.

	Fine-Tuning	Optimizer	Learning Rate	Batch Normalization	Dense 512 Units	Dense 256 Units	Dense 128 Units
**VGG16**	Last 8	SGD	0.001	✓	✓	-	-
**VGG19**	Last 8	SGD	0.001	✓	✓	-	-
**ResNet50V2**	Last 8	Adam	0.001	-	✓	-	-
**ResNet101V2**	Last 8	Adam	0.001	-	✓	-	-
**ResNet152V2**	Last 8	Adam	0.0001	-	✓	-	-
**InceptionV3**	Last 8	Adam	0.0001	-	✓	-	-
**MobileNetV2**	Last 8	SGD	0.0001	-	✓	-	-
**NASNet**	Last 8	Adam	0.0001	-	✓	-	-
**DenseNet121**	Last 8	Adam	0.0001	-	✓	-	-
**Xception**	Last 8	Adam	0.0001	-	✓	-	-

**Table 3:** Number of training, validation, and test images for each dataset in each randomized split of the stratified shuffle split cross-validation scheme.

Dataset	Total	Training	Validation	Test
Original Binary	400	280	40	80
Augmented Binary	1200	840	120	240
Original Multi-class	1500	1050	150	300
Augmented Multi-class	4500	3150	450	900

**Table 4:** Average class precision and recall, with standard deviation, on the original binary dataset. The metrics are computed over the 10 splits of the stratified shuffle split cross-validation scheme. The best values are highlighted in bold.

	No Defect		Defect	
	Precision	Recall	Precision	Recall
**VGG16**	85.26 ± 4.08%	79.00 ± 9.23%	81.03 ± 6.48%	86.00 ± 5.15%
**VGG19**	85.01 ± 6.89%	81.25 ± 8.16%	82.71 ± 6.58%	84.50 ± 9.60%
**ResNet50V2**	87.68 ± 5.60%	77.75 ± 7.37%	80.26 ± 5.84%	89.00 ± 4.77%
**ResNet101V2**	**89.89 ± 12.43%**	60.00 ± 17.64%	71.18 ± 8.56%	88.25 ± 21.51%
**ResNet152V2**	82.36 ± 4.79%	78.25 ± 6.23%	79.54 ± 4.13%	82.75 ± 6.37%
**InceptionV3**	83.86 ± 5.87%	73.25 ± 10.31%	76.80 ± 6.94%	85.50 ± 6.60%
**MobileNetV2**	83.83 ± 4.85%	75.75 ± 5.13%	77.96 ± 3.26%	85.00 ± 5.81%
**NASNet**	84.97 ± 5.64%	76.25 ± 7.00%	78.62 ± 5.32%	86.25 ± 5.94%
**DenseNet121**	87.22 ± 4.62%	85.25 ± 3.61%	85.60 ± 3.04%	87.25 ± 5.41%
**Xception**	89.57 ± 2.69%	**87.64 ± 4.70%**	**87.00 ± 5.68%**	**89.75 ± 3.05%**

**Table 5:** Average class precision and recall, with standard deviation, on the augmented binary dataset. The metrics are computed over the 10 splits of the stratified shuffle split cross-validation scheme. The best values are highlighted in bold.

	No-Defect		Defect	
	Precision	Recall	Precision	Recall
**VGG16**	93.71 ± 2.10%	93.00 ± 3.01%	93.10 ± 2.82%	93.75 ± 2.12%
**VGG19**	94.45 ± 3.69%	93.42 ± 3.34%	93.58 ± 2.97%	94.33 ± 4.15%
**ResNet50V2**	94.92 ± 3.40%	94.75 ± 2.91%	94.86 ± 2.56%	94.75 ± 3.93%
**ResNet101V2**	92.58 ± 3.14%	94.00 ± 3.09%	94.00 ± 2.87%	92.33 ± 3.45%
**ResNet152V2**	92.43 ± 2.08%	91.67 ± 2.86%	91.82 ± 2.43%	92.42 ± 2.37%
**InceptionV3**	94.44 ± 4.12%	91.33 ± 3.21%	91.62 ± 3.01%	94.50 ± 4.38%
**MobileNetV2**	**96.88 ± 1.17%**	95.42 ± 1.50%	95.50 ± 1.43%	**96.92 ± 1.18%**
**NASNet**	94.36 ± 2.42%	92.33 ± 2.20%	92.53 ± 1.98%	94.42 ± 2.58%
**DenseNet121**	96.82 ± 2.43%	**97.33 ± 1.66%**	**97.33 ± 1.63%**	96.75 ± 2.59%
**Xception**	96.63 ± 1.37%	97.17 ± 1.55%	97.18 ± 1.50%	96.58 ± 1.46%

**Table 6:** Average class precision and recall, with standard deviation, on the multi-class original dataset. The metrics are computed over the 10 splits of the stratified shuffle split cross-validation scheme. The best values are highlighted in bold.

	No Defect		Recoverable		Non-Recoverable	
	Precision	Recall	Precision	Recall	Precision	Recall
**VGG16**	88.05 ± 2.52%	**90.80 ± 2.96%**	**89.93 ± 4.36%**	**91.10 ± 3.11%**	**93.00 ± 3.12%**	88.50 ± 4.50%
**VGG19**	**88.89 ± 3.39%**	89.40 ± 4.50%	89.70 ± 3.76%	89.40 ± 3.80%	91.95 ± 3.78%	91.30 ± 2.57%
**ResNet50V2**	82.61 ± 5.45%	61.60 ± 21.20%	76.31 ± 7.41%	79.70 ± 9.58%	76.09 ± 12.47%	88.50 ± 2.69%
**ResNet101V2**	79.19 ± 3.94%	74.80 ± 11.04%	80.93 ± 6.92%	77.80 ± 5.36%	81.38 ± 5.39%	87.60 ± 5.68%
**ResNet152V2**	74.03 ± 4.89%	72.60 ± 12.03%	83.20 ± 7.15%	69.90 ± 6.49%	72.96 ± 9.34%	84.00 ± 6.13%
**InceptionV3**	82.21 ± 3.83%	79.60 ± 2.73%	83.27 ± 1.90%	83.60 ± 4.76%	86.21 ± 2.72%	88.20 ± 2.44%
**MobileNetV2**	78.01 ± 3.18%	88.60 ± 3.01%	85.14 ± 3.03%	83.00 ± 3.19%	91.00 ± 2.78%	80.60 ± 5.10%
**NASNet**	78.76 ± 3.02%	77.50 ± 3.61%	84.52 ± 2.99%	83.70 ± 3.16%	83.04 ± 2.52%	84.70 ± 3.55%
**DenseNet121**	86.02 ± 2.85%	87.10 ± 3.48%	89.90 ± 3.02%	88.40 ± 2.80%	91.37 ± 3.53%	**91.40 ± 2.94%**
**Xception**	83.92 ± 3.95%	87.10 ± 3.70%	87.57 ± 2.41%	87.30 ± 3.72%	90.46 ± 3.79%	86.90 ± 2.88%

**Table 7:** Average class precision and recall, with standard deviation, on the multi-class augmented dataset. The metrics are computed over the 10 splits of the stratified shuffle split cross-validation scheme. The best values are highlighted in bold.

	No Defect		Recoverable		Non-Recoverable	
	Precision	Recall	Precision	Recall	Precision	Recall
**VGG16**	95.54 ± 1.36%	**97.93 ± 0.94%**	**97.96 ± 1.00%**	**96.77 ± 1.21%**	96.80 ± 1.11%	95.50 ± 1.39%
**VGG19**	95.29 ± 1.51%	97.13 ± 1.14%	97.17 ± 0.88%	95.93 ± 1.39%	96.61 ± 0.88%	95.93 ± 1.67%
**ResNet50V2**	89.20 ± 3.61%	85.93 ± 6.42%	88.28 ± 5.15%	90.97 ± 2.48%	90.23 ± 4.49%	89.87 ± 6.23%
**ResNet101V2**	87.27 ± 3.72%	85.80 ± 9.26%	91.78 ± 4.46%	87.97 ± 3.84%	87.81 ± 4.97%	91.97 ± 3.63%
**ResNet152V2**	89.37 ± 3.36%	86.93 ± 4.78%	89.07 ± 2.96%	90.47 ± 2.81%	89.84 ± 4.84%	90.17 ± 4.32%
**InceptionV3**	93.51 ± 1.77%	95.13 ± 1.28%	95.78 ± 0.61%	94.60 ± 1.55%	94.96 ± 1.44%	94.43 ± 1.41%
**MobileNetV2**	91.05 ± 0.79%	94.63 ± 1.26%	95.45 ± 1.31%	92.70 ± 1.33%	94.21 ± 1.78%	93.17 ± 1.34%
**NASNet**	90.97 ± 1.20%	92.47 ± 2.14%	93.38 ± 1.57%	91.20 ± 0.56%	92.71 ± 1.33%	93.27 ± 1.21%
**DenseNet121**	**95.85 ± 1.38%**	97.20 ± 0.78%	97.81 ± 0.91%	96.30 ± 1.22%	**97.31 ± 0.96%**	**97.40 ± 1.10%**
**Xception**	94.08 ± 1.72%	96.77 ± 0.88%	97.32 ± 1.24%	95.83 ± 1.54%	96.33 ± 1.24%	94.97 ± 1.57%

## Data Availability

The dataset used in this study is publicly available at https://github.com/airtlab/surface-defect-classification-in-carbon-look-components-dataset (accessed on 10 July 2023). The source code of the experiments performed on such dataset is publicly available at https://github.com/airtlab/surface-defect-classification-in-carbon-look-components-using-deep-neural-networks (accessed on 10 July 2023).
